# Urinary Tetrahydrocannabinol After 4 Weeks of a Full-Spectrum, High-Cannabidiol Treatment in an Open-label Clinical Trial

**DOI:** 10.1001/jamapsychiatry.2020.3567

**Published:** 2020-11-04

**Authors:** M. Kathryn Dahlgren, Kelly A. Sagar, Ashley M. Lambros, Rosemary T. Smith, Staci A. Gruber

**Affiliations:** 1 Cognitive and Clinical Neuroimaging Core, McLean Hospital Imaging Center, Belmont, Massachusetts; 2 Marijuana Investigations for Neuroscientific Discovery Program, McLean Hospital Imaging Center, Belmont, Massachusetts; 3 Department of Psychiatry, Harvard Medical School, Boston, Massachusetts

## Abstract

This cohort study examines whether consumption of a high-cannabidiol product resulted in detectable amounts of Δ9-tetrahydrocannabinol metabolites in the urine samples of participants.

Despite the growing popularity of cannabidiol (CBD) products, specifically those derived from legal industrial hemp sources,^1^ few studies have directly assessed whether the use of high-CBD products could yield positive results on urinary drug tests assessing cannabis use through the detection of Δ9-tetrahydrocannabinol (Δ9-THC) metabolites. A recent short-term administration study found that a single exposure to vaporized CBD-dominant cannabis flower (CBD, 10.5%; Δ9-THC, 0.39%), which the authors noted was similar to hemp, resulted in positive drug test results (>15 ng/mL) for 2 of 6 participants within 4 to 8 hours of administration.^2^ However, to our knowledge, no studies have examined drug test results in those consistently using full-spectrum (ie, Δ9-THC–containing) CBD products. Accordingly, as part of an open-label clinical trial (NCT02548559) examining the use of a full-spectrum high-CBD product for anxiety (with unpublished results as yet), we monitored THC urinary drug status.

## Methods

This study was approved by the Partners Healthcare institutional review board, and all participants provided written informed consent. Study enrollment was conducted at McLean Hospital between June 2018 and February 2020. Participants were required to be 18 years or older, report at least moderate levels of anxiety assessed using well-validated measures,^3,4^ and test negative at baseline for 11-nor-9-carboxy-Δ^9^-tetrahydrocannabinol (THC-COOH), a major metabolite of Δ9-THC. Patients did not use cannabis and could not use any other cannabis/cannabinoid–based products throughout the 4-week trial. Women were required to have a negative pregnancy test result. Exclusion criteria included serious medical illness (eg, kidney or liver disease, neurological disorder). The open-label phase was capped at 15 participants to determine dosing and tolerability. The CONSORT guidelines were followed. A protocol is available in the Supplement.

The study product was formulated using a full-spectrum, high-CBD extract containing 9.97 mg/mL of CBD (1.04%) and 0.23 mg/mL of Δ9-THC (0.02%), as confirmed by ProVerde Laboratories. Patients self-administered 1 mL of the study product sublingually 3 times per day, for a targeted daily dose of approximately 30 mg of CBD and less than 1 mg of Δ9-THC. The actual dosage was quantified using outgoing vs incoming bottle weights, cross-referenced with weekly drug diaries. Urine drug assays (a 12-panel test, waived by the Clinical Laboratory Improvement Amendments^5^) assessed the presence of THC-COOH, which was confirmed via gas chromatography–mass spectrometry (Quest Diagnostics). Exploratory logistic regression analyses (SPSS version 25 [IBM]; α = .05, 2-tailed) assessed associations between THC-positive status, demographic variables, and creatinine, which is reflective of kidney function and hydration.

## Results

Of 15 patients enrolled (11 women [79%]; 12 White individuals [86%]), 1 discontinued participation because of use of another cannabinoid product; the remaining 14 patients completed all study procedures (Figure). The study drug was well tolerated; no serious adverse events were reported, and no patients reported psychoactivity. Patients used a mean (SD) of 3.48 (0.60) mL of the study product per day, equivalent to a mean (SD) of 34.73 (6.03) mg of CBD per day and 0.80 (0.14) mg of Δ9-THC per day. Results revealed that after 4 weeks, 7 participants (50%) tested positive for THC-COOH, while 7 tested negative. Gas chromatography–mass spectrometry results confirmed assay findings but indicated that the drug screen was often more sensitive than its stated lower limit of detection (50 ng/mL). Participants’ THC status was only significantly associated with creatinine levels (B, 1.92; *P* < .001; Table).

**Figure.**
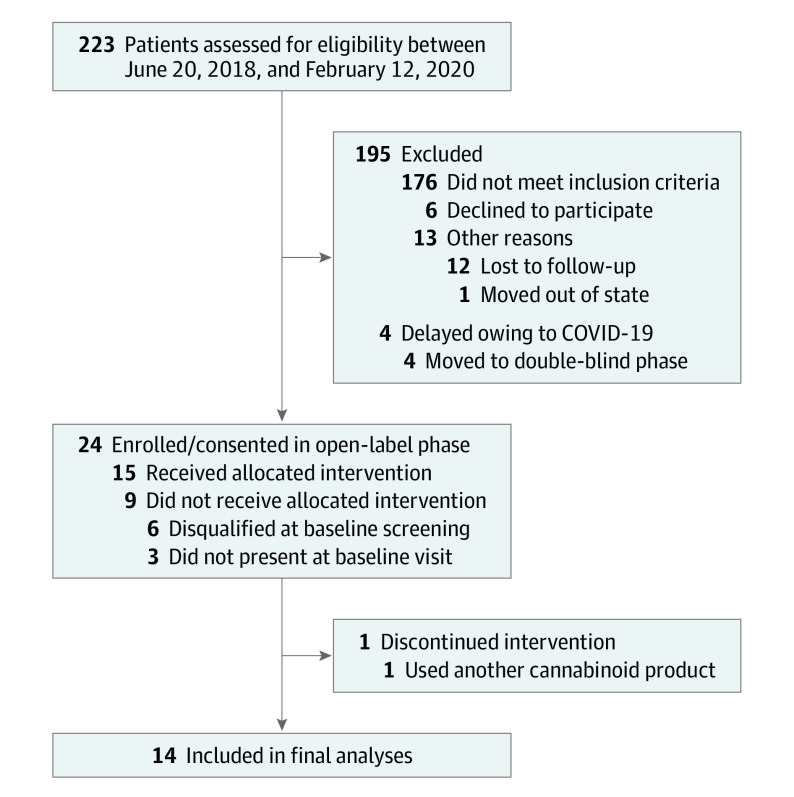
Study Recruitment and Enrollment CONSORT flowchart of recruitment and enrollment for the open-label phase of clinical trial NCT02548559. COVID-19 indicates coronavirus disease 2019.

**Table.** Demographics, Creatinine Levels, Product Use, and Δ9-Tetrahydrocannabinol Metabolite Results Following 4 Weeks of Treatment With a Full-Spectrum, High-Cannabidiol Product^a^

Participant No.	Age, decade	Education, y	BMI	Creatinine quantification by GC-MS, mg/dL	Mean product use, mL/d^b^	Urinary THC metabolite assay (THC-COOH)	
						Test result^c^	GC-MS quantification, ng/mL^d^
**Individual-level data**							
1	60s	19	26.30	21.50	2.32	Negative	BLQ
2	50s	12	22.30	25.70	2.69	Negative	BLQ
3	20s	16	24.80	48.30	2.91	Negative	BLQ
4	40s	18	31.63	128.56	3.19	Positive	13.10
5	60s	16	27.88	NA	3.34	Negative	NA
6	40s	16	22.46	264.81	3.37	Positive	71.50
7	20s	17	24.54	141.03	3.48	Positive	33.00
8	20s	16	20.60	53.33	3.48	Negative	8.00
9	20s	16	26.57	126.00	3.67	Positive	63.00
10	60s	18	25.82	90.22	3.69	Negative	8.30
11	60s	12	24.95	212.50	3.84	Positive	34.00
12	30s	15	30.11	107.14	3.96	Positive	30.00
13	20s	16	20.52	146.51	4.12	Positive	43.00
14	30s	18	34.54	35.70	4.70	Negative	BLQ
**Summary data**							
Total No. (%)	NA	NA	NA	NA	NA	7 positive: 7 negative (50:50)	NA
Mean (SD)	41.4 (16.9)	16.1 (2.1)	25.93 (4.07)	107.93 (73.54)	3.48 (0.60)	NA	35.99 (24.60)
**Univariate logistic regression results** ^e^							
B (*P* value)	−0.05 (.19)	−0.19 (.51)	−0.01 (.92)	1.92 (<.001)	1.17 (.28)	NA	NA
Odds ratio (95% CI)	0.95 (0.89-1.02)	0.83 (0.47-1.45)	0.99 (0.76-1.29)	6.80 (<0.01- 3.33 × 10^237^)	3.21 (0.40-26.12)	NA	NA

Abbreviations: BLQ, below the limit of quantification; BMI, body mass index (calculated as weight in kilograms divided by height in meters squared); GC-MS, gas chromatography–mass spectrometry; NA, not applicable; THC-COOH, 11-nor-9-carboxy-Δ^9^-tetrahydrocannabinol.

SI conversion factor: To convert creatinine to μmol/L, multiply by 88.4.

^a^ Individual ages are presented by decade to protect participants’ privacy and confidentiality. Individual-level data on participants’ self-selected race (via the established categories and definitions from the Race and Ethnic Standards for Federal Statistics and Administrative Reporting; 12 White individuals [86%]; 2 Black individuals [14%]) and sex (11 women [79%]; 3 men [21%]) are omitted to protect privacy and confidentiality. None of these variables had a significant association with urinary THC-COOH status.

^b^ Participants are arranged in ascending order by the mean amount of product used.

^c^ Lower limit of detection of THC-COOH, 50 ng/mL.

^d^ One sample could not be verified via GC-MS; descriptive statistics are provided for samples with detectable levels of THC.

^e^ With urinary THC-COOH status as the dependent variable.

## Discussion

The results suggest that patients consistently using full-spectrum, hemp-derived products may have positive test results for THC-COOH on a urinary drug screen. Studies with larger sample sizes are needed to more thoroughly assess which variables (product use, body mass index, age, sex, race, medication use, etc) contribute to positive findings in only some individuals, particularly those with higher creatinine levels. Importantly, the study product contained 0.02% of Δ9-THC by weight; in the US, hemp-derived products can legally contain 0.30% or less of Δ9-THC by weight, more than 10 times the amount of Δ9-THC as the current study product.

Despite limitations in sample size and diversity, these findings have important public health implications. It is often assumed individuals using hemp-derived products will test negative for THC. Current results indicate this may not be true, especially if assays are more sensitive than advertised, underscoring the potential for adverse consequences, including loss of employment and legal or treatment ramifications, despite the legality of hemp-derived products.

## Notes

Supplement.
Protocol.
