# Supplementary material for: Urinary Tetrahydrocannabinol After 4 Weeks of a Full-Spectrum, High-Cannabidiol Treatment in an Open-label Clinical Trial
Source: JAMA Psychiatry. 2020 Nov 4;78(3):335–7. doi: 10.1001/jamapsychiatry.2020.3567 (PMC9429743; doi:10.1001/jamapsychiatry.2020.3567)
Supplement: Supplement. — Protocol. [file YLD200012supp1_prod.pdf]

MCLEAN HOSPITAL RESEARCH PROTOCOL

1. Title: Sublingual Cannabidiol for Anxiety: Open-Label Phase

2. Investigators

Principal Investigator: Staci A. Gruber, Ph.D.
Cognitive and Clinical Neuroimaging Core
Neuroimaging Center
McLean Hospital
115 Mill Street
Belmont, MA 02478

Co-Investigators: Scott Lukas, Ph.D.
Behavioral Psychopharmacology Research Laboratory
Neuroimaging Center
McLean Hospital
115 Mill Street
Belmont, MA 02478

David P. Olson, MD, PhD.
Neuroimaging Center
McLean Hospital
115 Mill Street
Belmont, MA 02478

Consulting Clinicians: Milissa Kaufman, MD, PhD.
Dissociative Disorders and Trauma Program
The Hill Center for Women
McLean Hospital
115 Mill Street
Belmont, MA 02478

Franca Centorrino, MD
Schizophrenia and Bipolar Disorder Outpatient Clinic
Psychopharmacology Research Program
McLean Hospital
115 Mill Street
Belmont, MA 02478

Study Coordinators: Mary Kathryn Dahlgren, Ph.D.
Kelly A. Sagar, Ph.D.

Rosemary Smith, B.S.
Ashley Lambros, B.S.
Madeline Kuppe, B.S.
Celine El-Abboud, B.A.

3. Site where the study will be performed

Subjects will complete study visits at the Neuroimaging Center, McLean Hospital.

4. Introduction: Background and Significance

Cannabis has been used for medicinal purposes across many cultures for a range of disorders dating as far back as 2700 B.C. The plant is comprised of a variety of components, including phytocannabinoids that act on CB1 and CB2 receptors. Numerous phytocannabinoids are present in cannabis, including the major psychoactive constituent of cannabis, delta-9 tetrahydrocannabinol (THC), which acts as a CB1 receptor agonist. Another phytocannabinoid, cannabidiol (CBD), is a major non-psychoactive constituent of cannabis and is only a partial agonist at CB1 receptors. Increasing evidence indicates that CBD in particular may have significant medicinal properties and benefits; experimental studies in both animals and humans have demonstrated that CBD can act as an anticonvulsant, antipsychotic, and muscle relaxant. CBD is often found in higher levels in products dispensed as medical marijuana relative to strains used primarily for recreational use. Several studies have demonstrated that CBD produces acute anxiolytic effects in animals and humans, although thus far no clinical trials of CBD have been conducted in patients with anxiety. As a growing number of states are legalizing medical marijuana, a gap exists in the scientific literature regarding the effects of CBD on anxiety.

Rationale

Pilot data from the first phase of the MIND project, an observational study of the impact of medical marijuana on cognition, brain function, and quality of life, suggest an increase in quality of life measures accompanied by decreases in depression and anxiety following treatment with medical marijuana. Despite the recent interest in medical marijuana and cannabinoid-based products, no published studies to date have conducted a clinical trial of products high in CBD in individuals who suffer from anxiety. Further, none have systematically evaluated baseline and follow-up clinical state and related quality of life measures in individuals taking CBD, or assessed measures of brain structure and function before and after treatment with CBD using neuropsychological measures or neuroimaging. As a growing number of states are legalizing medical marijuana and increasing evidence suggests that CBD may exert anxiolytic effects, there is a gap in the literature regarding the effects of CBD, often found in higher levels in medical marijuana than recreational marijuana, on anxiety.

5. Research Objectives and Goals: Specific Aims

The proposed investigation is designed to examine the impact of the administration of a sublingual high-CBD compound on individuals with symptoms of anxiety.

Specific Aim 1: To assess pre- and post-CBD treatment clinical state ratings of anxiety and quality-of-life ratings in individuals with anxiety disorders.

Specific Aim 2: To assess pre- and post-CBD treatment performance on a range of neurocognitive measures designed to examine cognitive function.

Specific Aim 3: In a subset of individuals, to examine structural and functional changes that may occur in the brain following treatment with CBD using multimodal magnetic resonance imaging (MRI) techniques.

Exploratory Aim 1: In a subset of individuals, to examine the pharmacokinetics and pharmacodynamics (PK/PD) of this CBD tincture via continuous blood draw.

Exploratory Aim 2: to examine urinary THC status (positive/negative) and related variables over the course of treatment

6. Study Design, Procedures, and Subjects

Study Overview

This study is four-week, open label clinical trial of a high-CBD containing compound in individuals with anxiety. Participants will be pre-screened by phone in order to evaluate their eligibility for the study. If approved, participants will come to the hospital for a baseline/screening visit, and will complete a structured clinical interview, clinical and quality of life questionnaires, and cognitive assessments. Enrolled participants will be given a solution to use for the duration of the study; participants will be instructed to self-administer 1 milliliter (ml) of the solution under the tongue three times per day for four weeks. Throughout the treatment period, participants will return to the hospital on a weekly basis to complete questionnaires about their mood and quality of life. Participants will also return to the hospital for a final visit after four weeks of treatment to complete additional questionnaires and cognitive assessments. All patients will also complete in-house drug assays and positive results will be confirmed by an outside laboratory (Quest Diagnostics).

The open-label phase I trial will assess the efficacy of the dose chosen for a subsequent double-blind phase of the study. This open-label trial will enroll up to 16 participants who have expressed interest in using CBD, and who have anxiety. A subset of participants (up to n=16) will also complete an extra visit that includes clinical rating scales and a 2-hour continuous blood draw directly following administration of the tincture in order to assess plasma concentration of CBD over time and correlation with anxiety.

Study Visits

Subjects will meet with the Principal Investigator, a Clinical Neuropsychologist, or a trained Research Assistant at McLean Hospital for five visits (baseline, and four weekly visits throughout the 4-week treatment period), each lasting approximately 1 to 3 hours. A subset of subjects in the open-label phase will also complete an optional 6th visit; this visit will occur after a >1 week washout period after the final (week 4) visit, where they will return to the lab to complete a 2-hour continuous blood draw after administration of the study product. Subjects will

review and sign the approved informed consent form prior to engaging in any study procedures. A structured clinical interview (SCID-P) will be administered, and demographic information, substance abuse/use, and medical histories will also be obtained and reviewed with the study physician. Study inclusion/exclusion criteria will be applied, and appropriate subjects will be enrolled and complete the rest of the study visit.

A tiered payment system will be used for a payment of $75 at visit 1, $50 at visits 2-4, and $75 at visit 5 for a total of $300; if termination occurs during any point of the visit, subjects are compensated at a rate of $25 per hour.

Outcome Measures

Primary Outcome Measures:

1. Change from Baseline in Self-Reported Anxiety as Assessed by the Beck Anxiety Inventory (BAI) [Time Frame: Week 1, Week 2, Week 3, Week 4]

The BAI is a 21-item self-report measure used to rate subjective, somatic, and panic-related symptoms of anxiety on a scale of 0 to 3, and will be given to participants on a weekly basis.

Secondary Outcome Measures:

1. Change from Baseline in Anxiety Assessed by the Hamilton Anxiety Scale (HAM-A) [Time Frame: Week 1, Week 2, Week 3, Week 4]

The HAM-A is an administered measure of anxiety that will be given on a weekly basis; a variety of symptoms are rated on a scale of 0 to 4.

2. Change from Baseline in Self-Reported Anxiety Assessed by the State-Trait Anxiety Inventory (STAI) [Time Frame: Week 1, Week 2, Week 3, Week 4]

This self-report measure is comprised of two 20-item scales, with a range of four possible responses from 1 to 4, and differentiates between the more temporary condition of "state" anxiety and the more general quality of "trait" anxiety. It will be given on a weekly basis.

Subjects

Subjects will be recruited through IRB-approved advertisements and flyers in regions that have approved medical marijuana. Additionally, medical marijuana certification and healthcare facilities throughout New England may also refer interested patients to contact the study recruitment line for further screening. These healthcare groups provide their interested patients who meet for general inclusion criteria with study recruitment materials.

Recruitment and Informed Consent

Participants will be recruited through IRB-approved advertisements and flyers throughout the Greater Boston area, including medical marijuana certification centers, as well as online advertisements (including the Partners Clinical Trials website). Written, informed consent will be obtained from all participants following a screening interview to determine eligibility. The consent form will include a description of the study, information about procedures, and assurances of confidentiality. Prior to signing the informed consent, subjects will be asked if they have any questions regarding the conduct or design of the study. A copy of the signed consent form will be given to the study subject, and a copy placed in their research record. All subjects will be reminded that their participation is completely voluntary, and may withdraw or discontinue the study at any time. The informed consent will be approved by the McLean Hospital Institutional Review Board, which monitors study progress, safety and outcome on a regular basis.

Confidentiality of information collected will be maintained with the assignment of an identification number or code, which will be used in place of subject names in all data analyses and reports. Computer systems are located in the Cognitive and Clinical Neuroimaging Core. Keys showing the assignment of identification numbers to subject names will be stored with subject files in room 204 of the Neuroimaging Building under lock and key. All of the data that is collected will be kept for a minimum of seven years once the study has been completed. Only the Principal Investigator, Dr. Staci Gruber and her research staff will have access to the data that is collected.

All subjects will be required to give informed consent and must understand all procedures prior to their participation in the study. A member of the research staff will explain the consenting procedure and be available for any questions that arise from the consent form. All subjects must be able to give their own consent for participation. All signed consent forms will be kept in the subject's case report form in room 204 of the Neuroimaging Center under lock and key.

Inclusion Criteria:
- 18 or older
- Native English speaker or acquired English prior to age 5
- Provides informed consent

Exclusion Criteria:
- Non-native English speakers
- Estimated IQ < 75
- Pregnancy
- Presence of serious medical illness, including liver or kidney disease, neurological disorder, or certain psychiatric disorders

Subjects will be asked to complete a 3-item demographics questionnaire in order for the researchers to obtain accurate information about subject's racial and ethnic identification. This information is required to be reported in federal grant progress reports.

No subjects will be excluded on the basis of race, sex, ethnicity or sexual orientation.

Study Termination Criteria

Subjects may withdraw at any time and for any reason. A subject's participation in the trial will end if any of the following criteria are met:

a) Completion of the study
b) Subject reports adverse effects of CBD and wishes to leave the study, or if study staff determine that study termination is appropriate.
c) Subject does not use CBD as directed
d) Subjects reports use of cannabis or other cannabinoid-containing products
e) Subject sustains a significant head injury
f) Any study exclusions are met (i.e. change in medical status, pregnancy, etc.)

Data Collection

Results from subjects' demographic, clinical and neuroimaging data will be coded with a subject identification number, evaluated, and kept under lock and key at the CCNC office in room 204, Neuroimaging Center, McLean Hospital; no personally identifiable information accompanies this data. Urine samples will be coded by subject's date of birth, initials, and study code.

Monitoring and Quality Assurance

In the unlikely event that an adverse event occurs, it will be reported to the primary investigator and the Institutional Review Board's guidelines will be followed to ensure adequate reporting and response. Regulatory binders are kept for all studies at McLean Hospital in order to constantly monitor investigations and ensure that all data is collected safely.

The principal investigator will be responsible for monitoring and ensuring the integrity of the data and adherence to the IRB-approved protocol. They will review any questions or concerns regarding data, and will review each signed consent form for the study. There are no plans to utilize a Data Safety Monitoring Board (DSMB) due to the extremely low side-effect profile of CBD, but if the IRB deems that an independent DSMB is necessary, the investigators would be happy to suggest individuals who are appropriate or to consider outside suggestions from IRB members.

Data analyses will be conducted prior to initiating the double-blind phase in order to ensure that patients derive clinical benefit from the dose selected. Specifically, in conjunction with Drs. Olson and Kaufman, we will assess clinical response after the first 5 patients have completed their 4 week trial. If we do not see clinical improvement based on a review of clinical scales, subjective reports and performance, we will consider increasing the dose to 1.5 droppers three times a day for a total of 45 mg CBD per day. Clinical improvement using scales will be defined as a 15% reduction in BAI scores from baseline. Findings will be used to inform the double blind phase of the study, which would not begin until adequate dose/response is achieved. An amendment would be submitted to the IRB for approval prior to adjusting the dose of CBD.

Statistical Approaches

All statistical analyses will be conducted using IBM SPSS Statistics (version 20). Data will be screened for outliers and any data points more than 2 standard deviations from the group means will be excluded. Additionally, data will be screened for skew, kurtosis, non-normality, and homogeneity of variance. If the assumptions of parametric inferential analyses are not met, the appropriate non-parametric analyses will be performed. Baseline demographic, clinical, and neuropsychological data will be assessed using 2-tailed analyses of variance (ANOVAs). Between-group treatment differences over the course of the study will be assessed using a mixed model ANOVAs with Scheffe post hoc tests. Exploratory Pearson's r correlations (2-tailed) will be utilized to determine whether THC positive/negative status is related to demographic variables and amount of product used.

Potential Risks and Discomforts

CBD Administration

The proposed investigation is low risk because subjects are not asked to alter their existing medication regimen and instead are simply "adding on" either a placebo or a high-CBD compound. CBD has been shown to have an extremely low side effect profile and since the total amount of THC will not exceed 0.3% by weight, we do not expect significant side effects or psychoactive effects. CBD is not a scheduled substance, and there is no risk of intoxication or addiction to CBD.

Sublingual tinctures are unlikely to be viewed by the public as analogous to recreational smoked marijuana, thus decreasing the risk of the subject experiencing any potential negative appraisal arising from perceived notions associated with marijuana use while partaking in this study.

As with any clinical trial, there are risks of experiencing side effects from the administration of CBD or placebo; these side effects are very rare. Cunha et al. (1980) reported no signs of toxicity or serious side effects; in this two-part, placebo-controlled, double blind study, healthy volunteers were given 3 mg/kg of CBD or placebo per day for 30 days, and patients with epilepsy were given 200-300mg CBD or placebo per day for 4.5 months. The total dose of CBD per day for the current study will be 30mg – thus, we expect no significant side effects. Other studies have reported no adverse effects of CBD in patients with Huntington's disease, schizophrenia, and Parkinson's disease after repeated administration (Consroe et al. 1991; Leweke et al. 2012; Zuardi et al. 2006 and 2009).

Additionally, the safety of CBD administration in pregnant women and fetuses is unknown. Female participants capable of child-bearing will be asked to provide a sample of urine before the study is begun and at each subsequent study visit in order to screen for pregnancy. If the pregnancy test is positive at any point during the study, the subject will be immediately disqualified and participation in the study will cease. Participation requires that the participant uses contraception methods (such as abstinence, diaphragm, condom, or an intrauterine device) to prevent pregnancy for the duration of the study. The participant will be asked to notify study staff immediately if she misses a period or thinks she might be pregnant. In this case, the participant may have to withdraw from the study.

Protection of risks: subjects will have the opportunity to report adverse effects at their weekly check-in visits via direct contact with the PI and study staff. This weekly reporting will reduce the likelihood that subjects experience significant negative side effects for any significant period of time, and the PI as well as they study physician will be reachable by page 24 hours a day 7 days a week.

Blood Draw

Subjects may have a bruise or pain from the site where the blood sample is acquired. There is also a small risk of feeling lightheaded, fainting, or infection. Clinical staff will be available to evaluate the subject if they experience any of these symptoms.

Potential Benefits

There may be no direct benefits to the subjects; however, based on previous research, it is reasonable to expect that some subjects may experience an improvement in clinical state or quality of life related to a reduction in anxiety.

It is reasonable to expect that this study will contribute to overall knowledge in this field and potentially provide benefits to society in general through improvements in understanding the effects of medicinal marijuana use, as well as the treatment of anxiety. Subjects may benefit from knowing that the results of this study may improve the future care of people with anxiety.

Compensation

Total compensation for completion of the entire study will be $350, broken down as follows: $75 for visits 1 and 5, $50 for each of three interim visits, a $50 completion bonus if the subject completes the full study. Subjects in the open-label phase who complete the optional 6th visit, which includes a blood draw, will receive an additional $150, for a total of $500. If subjects in the double-blind phase complete the additional MR scanning protocol at visits 1 and 5, they will receive an additional $75 for those two visits for a total additional payment of $150; therefore, the total compensation for those who complete the MR scanning component of the study will be $500. Subjects may withdraw from the study at any point; and will still receive payment for the portion of the study they completed at a rate of $25/hr.

For tax reporting purposes subjects' social security numbers are needed in order to process payment for participation in this study. McLean Hospital is required to inform the IRS of any payments received as a subject in research studies if they total over $600 in a given calendar year. If that occurs, subjects will receive a 1099 form at the end of the year. No information identifying why payment was received is communicated to either the Hospital's accounting department or the government. This information is kept strictly confidential and is not retained in research or medical records.

Anticipated Results and Potential Pitfalls

We anticipate acquiring neurocognitive, clinical state, quality of life, and sleep/activity data from individuals who have been diagnosed with anxiety disorders and treated with CBD or placebo.

Unsuccessful recruitment is a potential pitfall of this study. If subjects are not accurately screened or interviewed, their data may not be optimal for comparison.

Timeline

The initial phase of the project is expected to last approximately 18-24 months, with the possibility of extension and expansion after an initial assessment of pilot study findings. Initial study set-up should take no longer than 3 months, once funding is received, and subject recruitment should proceed immediately thereafter.
